# Supplementary material for: Healthy dynamics of CD4 T cells may drive HIV resurgence in perinatally-infected infants on antiretroviral therapy
Source: PLoS Pathog. 2022 Aug 15;18(8):e1010751. doi: 10.1371/journal.ppat.1010751 (PMC9410541; doi:10.1371/journal.ppat.1010751)
Supplement: S1 Text — A—List of clinical covariates. B—Additional information regarding infant adherence. C—Model. D—Structural Identifiability. E—Nonlinear Mixed Effects Modeling in Monolix. (PDF) [file ppat.1010751.s001.pdf]

Healthy dynamics of CD4 T cells may drive HIV resurgence in perinatally-infected infants on antiretroviral therapy

Sinead E. Morris[1], Renate Strehlau[2], Stephanie Shiau[3,4], Elaine J. Abrams[4,5,6],
Caroline T. Tiemessen[7], Louise Kuhn[3,4], Andrew J. Yates[1],
on behalf of the EPIICAL Consortium and the LEOPARD study team

SUPPORTING INFORMATION

S1 Text

A – List of clinical covariates

Table 1: Summary of the clinical covariates included in our statistical analyses.

Covariate	Group	N	Variable treatment*
Sex	Male	59	Categorical
	Female	63	
Preterm	Yes	88	Categorical
	No	103	
Delivery mode	Normal vaginal delivery	87	Categorical
	Caesarean section	35	
Birth weight (g)	<2500	29	Continuous
	2500+	93	
Age at ART initiation (days)	<2	49	Categorical
	2–14	56	
	14+	17	
Pre-treatment CD4 percentage	<35	33	Continuous
	35+	61	
	Not recorded	28	
Mother's viral load (copies ml^{-1})	<1000	32	Continuous
	1000+	90	
Mother's CD4 count (cells μl^{-1})	<350	61	Continuous
	350+	61	
Mother's CD4 percent	<25	77	Continuous
	25+	45	
Maternal prenatal ART history	None	25	Categorical
	Initiated 12+ weeks into pregnancy	37	
	Initiated <12 weeks into pregnancy	41	
	Initiated before pregnancy	18	
	Unknown	1	
Breastfeeding	Some	96	Categorical
	None	26	

*Variable treatment when testing for associations with resurgence, estimated model parameters, and/or adherence reports. For continuous variables, groups are included to qualitatively describe the covariate distribution but are not used in the analysis

B – Additional information regarding infant adherence

At each study visit, the infant's caregiver provided additional information for a questionnaire taken by the attending physician. Caregivers were asked if any doses had been missed since the previous visit and, if so, how many. They were also asked about any challenges administering the medication, including drug tolerance issues (e.g. if the infant spit up the medicine and repeat doses were required).

This information was a valuable supplement to estimates of adherence calculated from the amount of medication returned at the visit, given that these were often missing (due to leftover medication spilling or being left at home). If a particular adherence estimate was missing, we checked the corresponding questionnaire; if the physician noted serious adherence concerns for that drug, such as a series of missed doses, adherence was labeled as 'poor'. See S1 Figure for the resulting time series.

C – Model

Starting with the original set of equations,

$$\begin{aligned}
\frac{dT}{dt} &= \theta(t,T) - (1-\epsilon_1)\beta V_{\mathrm{I}} T \\
\frac{dI}{dt} &= \phi(1-\epsilon_1)\beta V_{\mathrm{I}} T - \delta I + a \\
\frac{dV_{\mathrm{I}}}{dt} &= (1-\epsilon_2)pI - cV_{\mathrm{I}} \\
\frac{dV_{\mathrm{NI}}}{dt} &= \epsilon_2 pI - cV_{\mathrm{NI}},
\end{aligned}$$

we assume that viral dynamics occur on a faster timescale than those of CD4 T cells, i.e. $dV_{\mathrm{I}}/dt = dV_{\mathrm{NI}}/dt = 0$. This gives $I = cV/p$ where the total virus $V = V_{\mathrm{I}} + V_{\mathrm{NI}}$, and $V_{\mathrm{I}} = (1-\epsilon_2)V$. We can then rewrite the above as

$$\begin{aligned}
\frac{dT}{dt} &= \theta(t,T) - (1-\epsilon_1)(1-\epsilon_2)\beta VT \\
\frac{c}{p}\frac{dV}{dt} &= \phi(1-\epsilon_1)\beta(1-\epsilon_2)VT - \frac{\delta cV}{p} + a,
\end{aligned}$$

or

$$\begin{aligned}
\frac{dT}{dt} &= \theta(t,T) - (1-\epsilon_1)(1-\epsilon_2)\beta VT \\
\frac{dV}{dt} &= \frac{\phi p}{c}\beta(1-\epsilon_1)(1-\epsilon_2)VT - \delta V + \frac{ap}{c}.
\end{aligned}$$

Setting $\beta_0 = \beta(1-\epsilon_1)(1-\epsilon_2)$ and $\bar{p} = p/c$ then gives the reduced system,

$$\begin{aligned}
\frac{dT}{dt} &= \theta(t,T) - \beta_0 VT \\
\frac{dV}{dt} &= \phi\,\bar{p}\beta_0 VT - \delta V + a\bar{p}.
\end{aligned}$$

D – Structural Identifiability

We explore the structural identifiability of the equations using the approach of Castro and de Boer (2020) [1]. First, we define scaling factors for all parameters we want to estimate, i.e. $u_{\beta_0}, u_{\bar{p}}, u_d, u_a$ and $u_{\bar{r}}$. The scaling factors for all fixed parameters are equal to one. Similarly, since both V and T are observed, we do not need to define any variable scaling factors.

Next, we equate all functionally independent terms in our equations that contain these parameters to their scaled counterparts. Assuming T_R and ϕ are fixed gives:

$$
\begin{aligned}
u_{\bar{r}}\bar{r} = \bar{r} \quad &\Leftrightarrow \quad u_{\bar{r}} = 1 \\
-u_{\beta_0}\beta_0 VT = -\beta_0 VT \quad &\Leftrightarrow \quad u_{\beta_0} = 1 \\
\phi u_{\bar{p}}\bar{p}u_{\beta_0}\beta_0 VT = \phi\bar{p}\beta_0 VT \quad &\Leftrightarrow \quad u_{\bar{p}} = 1 \quad \text{since} \quad u_{\beta_0} = 1 \\
-u_\delta \delta V = -\delta V \quad &\Leftrightarrow \quad u_\delta = 1 \\
u_a a u_{\bar{p}}\bar{p} = a\bar{p} \quad &\Leftrightarrow \quad u_a = 1 \quad \text{since} \quad u_{\bar{p}} = 1.
\end{aligned}
$$

Since all scaling factors have solution equal to 1, all estimated parameters are identifiable. Note that we assume r is identifiable if $\bar{r}$ is identifiable and T_R is fixed.

E – Nonlinear Mixed Effects Modeling in Monolix

We fit the following system of equations in Monolix,

$$\frac{dT}{dt} = \theta(t, T) - \beta_0 VT$$
$$\frac{dV}{dt} = \phi\,\bar{p}\beta_0 VT - \delta V + a\bar{p}.$$

All VL observations below the detection threshold of 20 copies ml^{-1} are treated as censored values. In line with previous pharmacokinetic and viral dynamics modeling, we assumed both $V(t)$ and $T(t)$ were lognormally distributed with combined error models, i.e. for variable $X_i(t)$, the residual error is expressed as the sum of a constant term and a term proportional to $X_i(t)$ ('combined1' in Monolix) [2–4].

We fixed b_0 and b_1 across infants to the values given in Table 1 in the text. Initially we fixed T_R = 222 days and $\phi = 0.35$ across all infants, but subsequently explored fits when these values are varied, and when they were freely estimated. All other parameters were estimated and assumed to have both fixed and random effects. Guided by exploratory fits, each estimated parameter was assumed to follow a lognormal distribution, with the exception of a which followed a logit-normal distribution between 0 and 0.1, and T_R, T_A which followed normal distributions (when estimated). Initial estimates for all population parameters are given in the Table below. Initial estimates for the residual error models were kept at their default values. Following exploratory fits, we allowed for a correlation between β_0 and d, but assumed all other parameters were independent.

Initial estimates for the population parameters in Monolix

Parameter	Distribution	Fixed effect	Standard Deviation of Random Effects
β_0	Lognormal	1×10^{-6}	0.1
$\bar{p}$	Lognormal	5000	1
δ	Lognormal	0.3	0.1
a	Logitnormal (0–0.1)	0.01	0.1
r	Logormal	9	0.25
$\phi^{\star}$	Lognormal	0.05–0.35	0.1
$T_R{}^{\star}$	Normal	210–235	2
$T_A{}^{\star}$	Normal	365	2
T_0	Lognormal	3500	1
V_0	Lognormal	30,000	1

*when estimated

F - Statistical comparison of models

T_R	ϕ	AIC difference
fixed	fixed	0.0
estimated (random effects)	fixed	32.1
estimated (random & fixed effects)	fixed	24.4
fixed	estimated (random effects)	25.1
fixed	estimated (random & fixed effects)	47.9

Comparing models with T_R and ϕ allowed to vary across infants. When fixed, T_R and ϕ took their best-fit values of 230 and 0.35, respectively (S3 Fig.). The AIC difference for model i was calculated as $\text{AIC}_i - \text{AIC}_{\text{min}}$, where AIC_{min} is the minimum AIC value across all models. The model with zero difference is the model with lowest AIC and thus is the most strongly favored.

References

1. Castro M, de Boer RJ. Testing structural identifiability by a simple scaling method. *PLOS Computational Biology*. 2020;16(11):1–15. doi:10.1371/journal.pcbi.1008248.

2. Prague M, Gerold JM, Balelli I, et al. Viral rebound kinetics following single and combination immunotherapy for HIV/SIV. *bioRxiv*. 2019;doi:10.1101/700401.

3. Monolix version 2020R1. Antony, France: Lixosoft SAS; 2020. Available from: `http://lixoft.com/products/monolix/`.

4. Mould D, Upton R. Basic Concepts in Population Modeling, Simulation, and Model-Based Drug Development – Part 2: Introduction to Pharmacokinetic Modeling Methods. *CPT: Pharmacometrics & Systems Pharmacology*. 2013;2(4):38. doi:10.1038/psp.2013.14.
