# Supplementary figures and images for: Healthy dynamics of CD4 T cells may drive HIV resurgence in perinatally-infected infants on antiretroviral therapy
Source: PLoS Pathog. 2022 Aug 15;18(8):e1010751. doi: 10.1371/journal.ppat.1010751 (PMC9410541; doi:10.1371/journal.ppat.1010751)

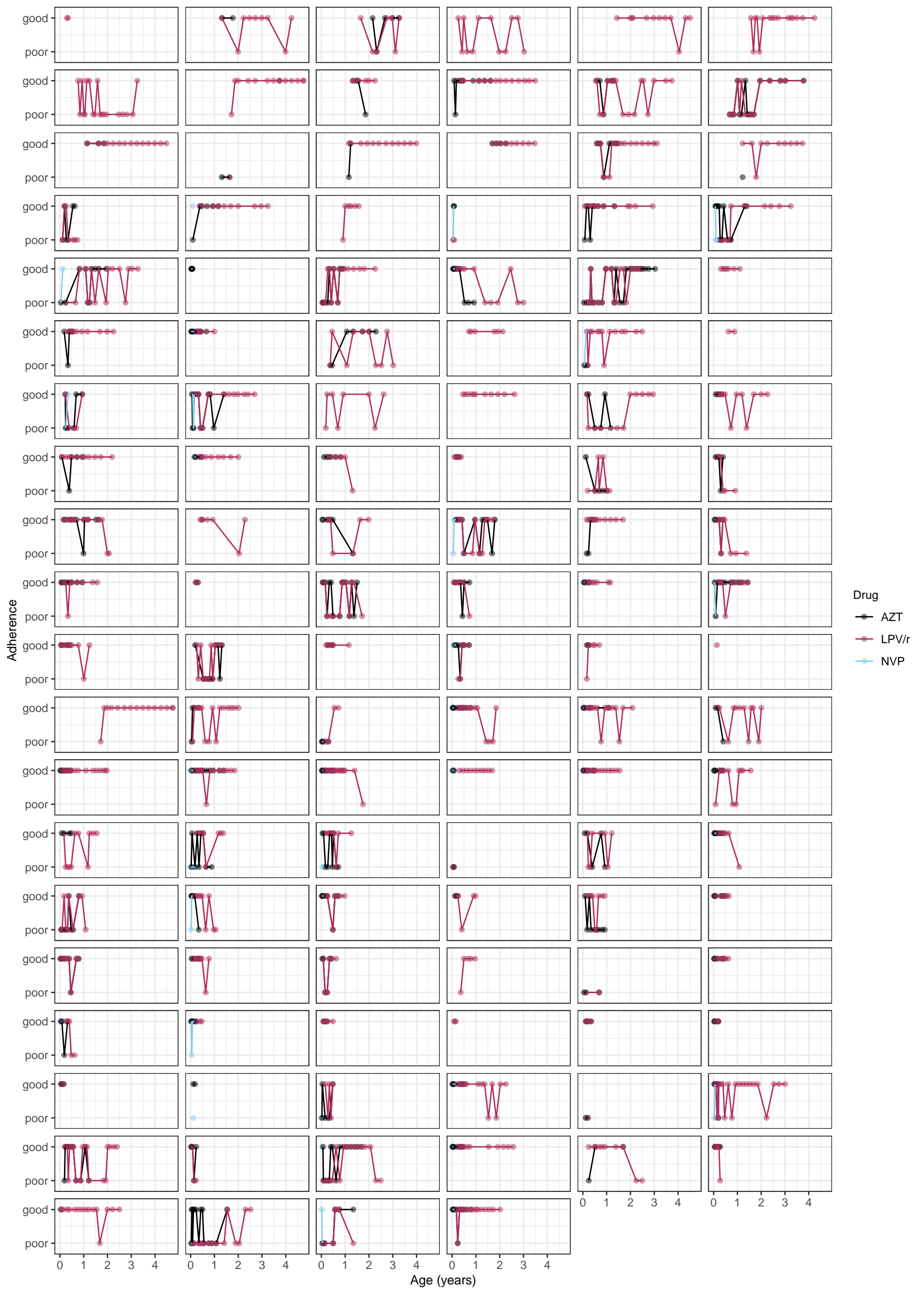

Supplement: S1 Fig — Adherence estimates greater than 90% were labeled ‘good’; and all others ‘poor’. Each panel represents a different infant. (PDF) [file ppat.1010751.s002.pdf]

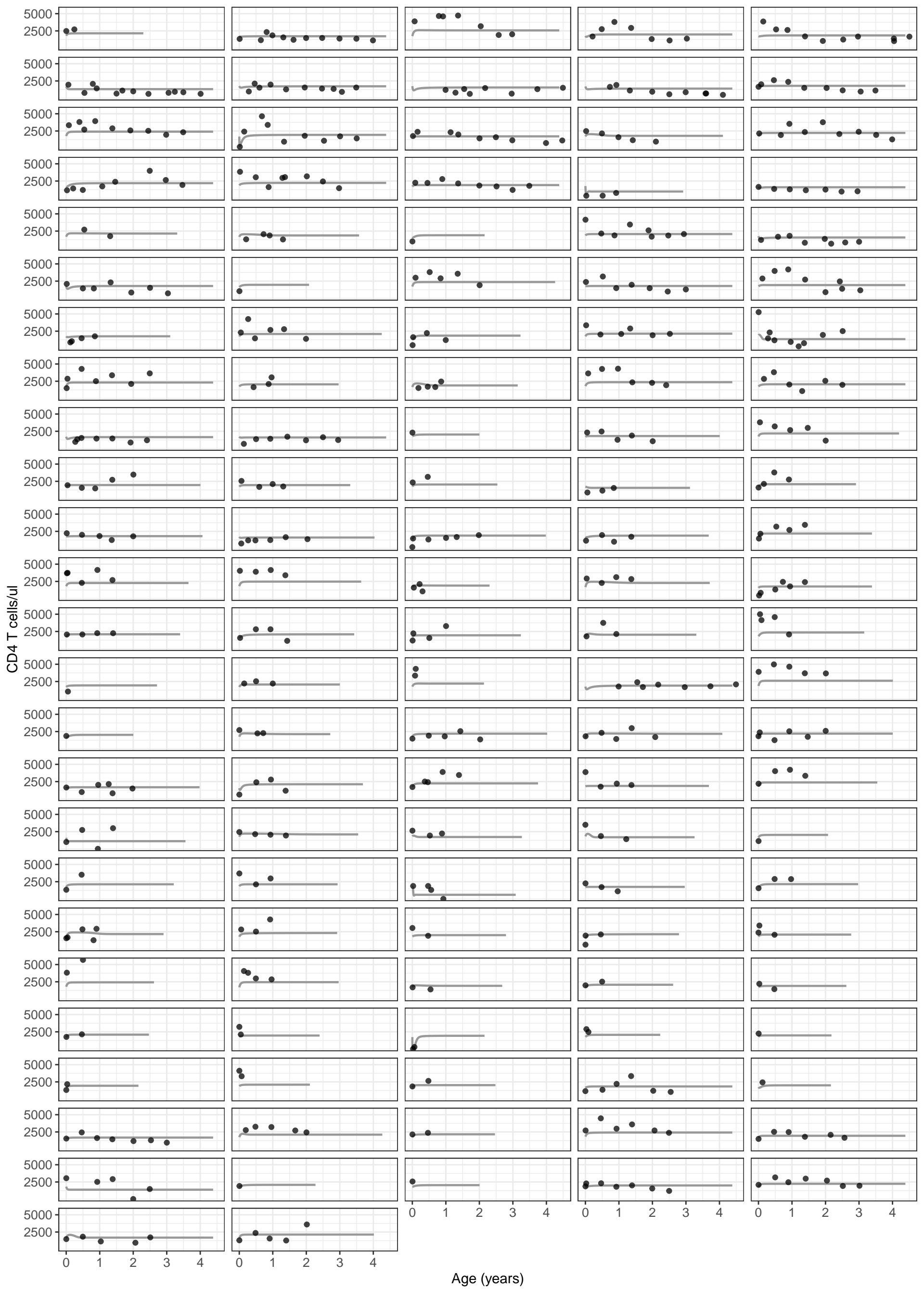

Supplement: S2 Fig — Each panel represents a different infant, points represent the data, and solid lines are the model fits. Here θ(t, T) = λ − dTT, with λ and dT assumed to have lognormal distributions. Initial estimates for the population mean were 1000 cells μl−1 day−1 and 0.25 day−1, respectively, and for the standard deviation were 1 and 0.1, respectively. (PDF) [file ppat.1010751.s003.pdf]

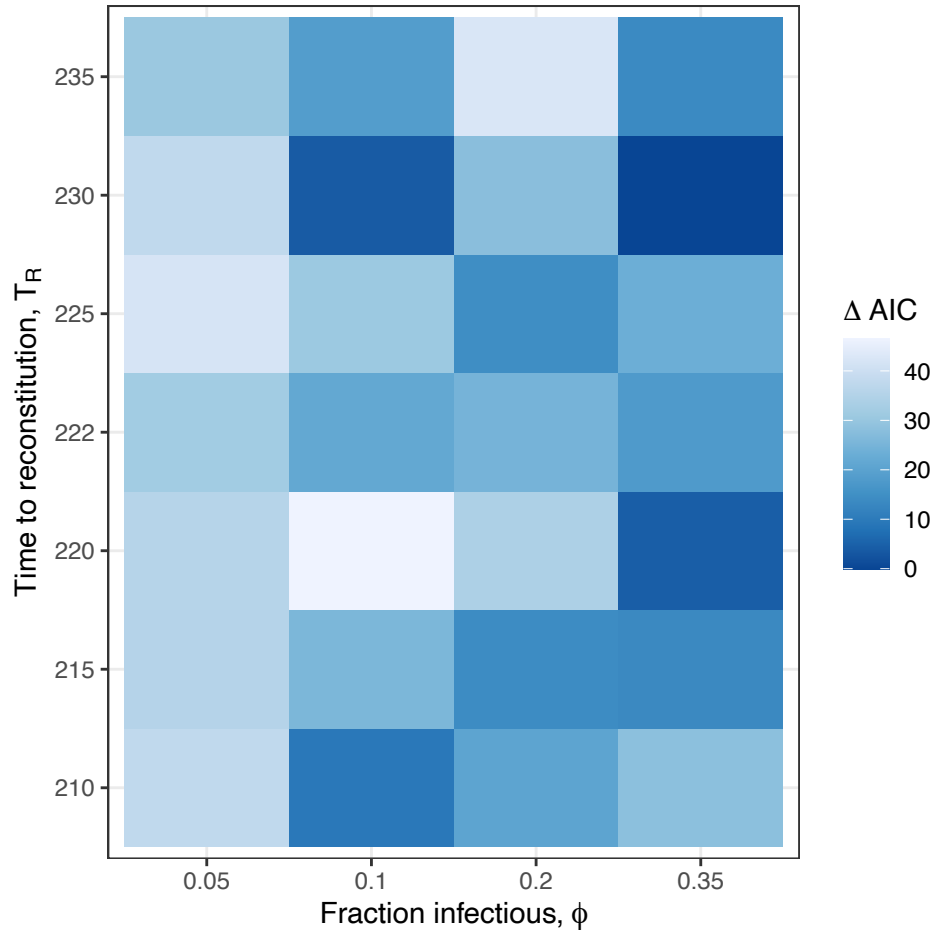

Supplement: S3 Fig — The AIC difference for model i was calculated as AICi− AICmin, where AICmin is the minimum AIC value across all models. The model with zero difference is the model with lowest AIC and thus is the most strongly favored. (PDF) [file ppat.1010751.s004.pdf]

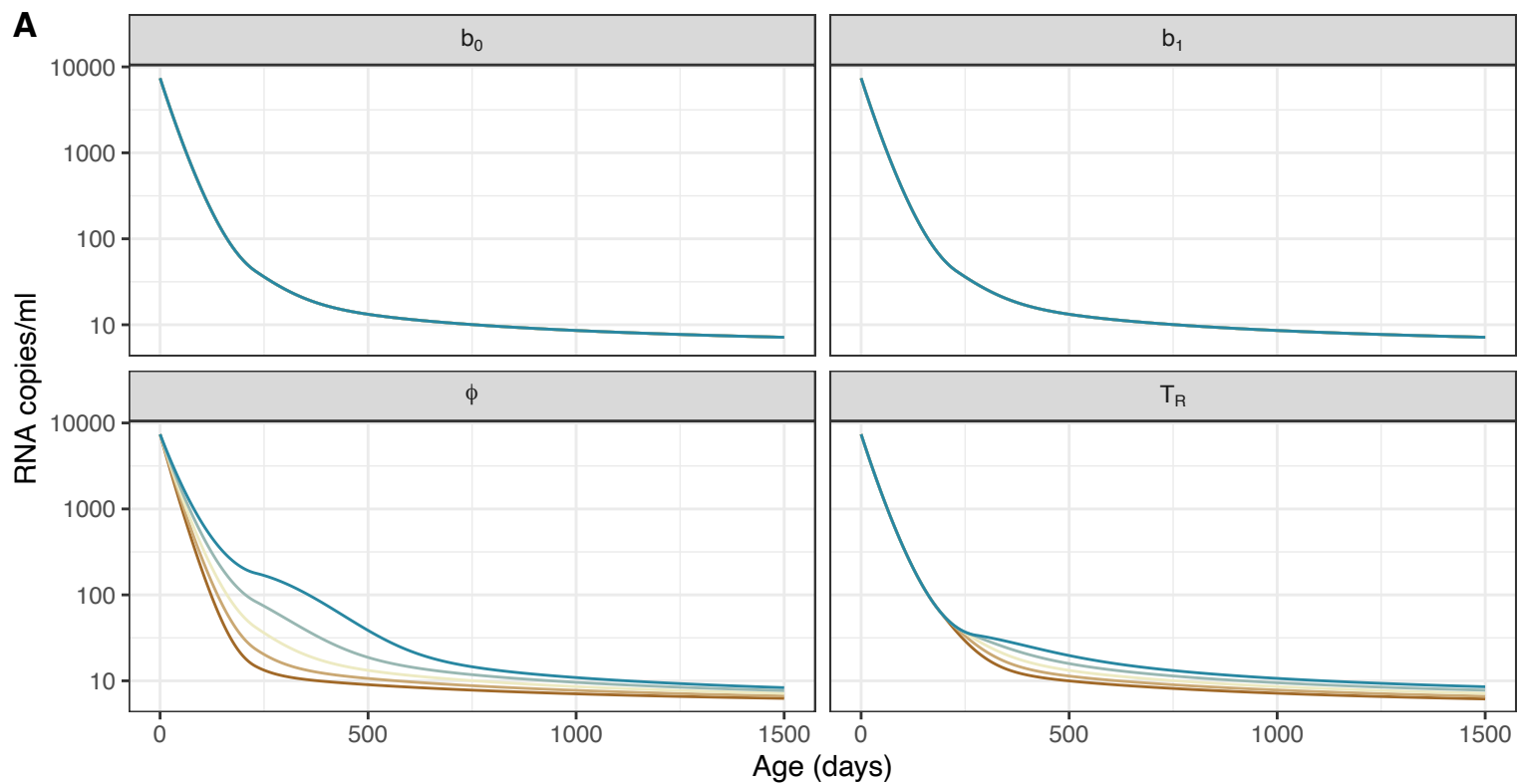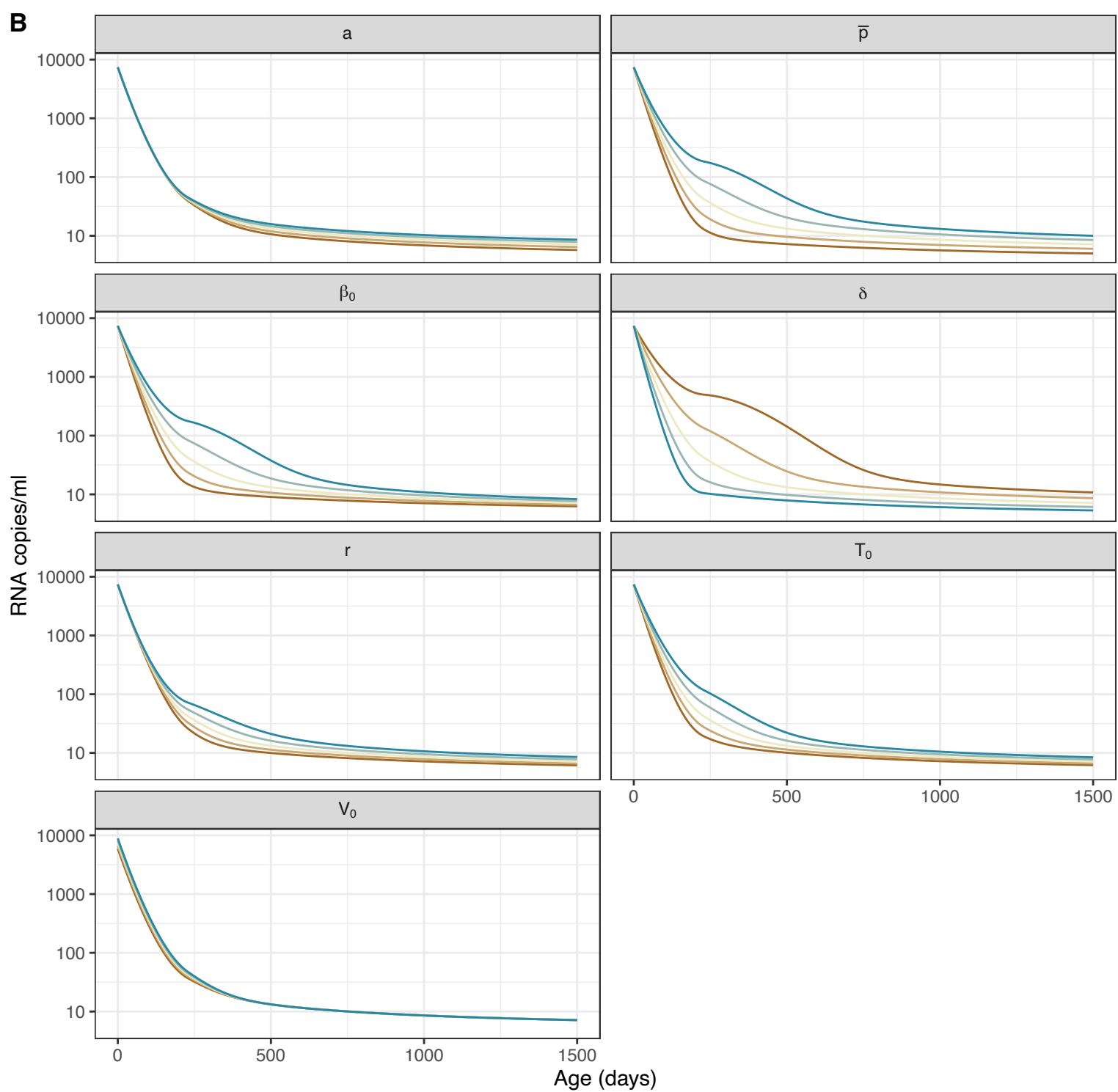

Change in estimated value — -20% — -10% — 0% — 10% — 20%

Supplement: S4 Fig — Each fixed (A) or estimated (B) parameter was varied within 20% of its original value while keeping all other parameters at their original values. Original values for the estimated parameters were the population-level means from the best-fit model. (PDF) [file ppat.1010751.s005.pdf]

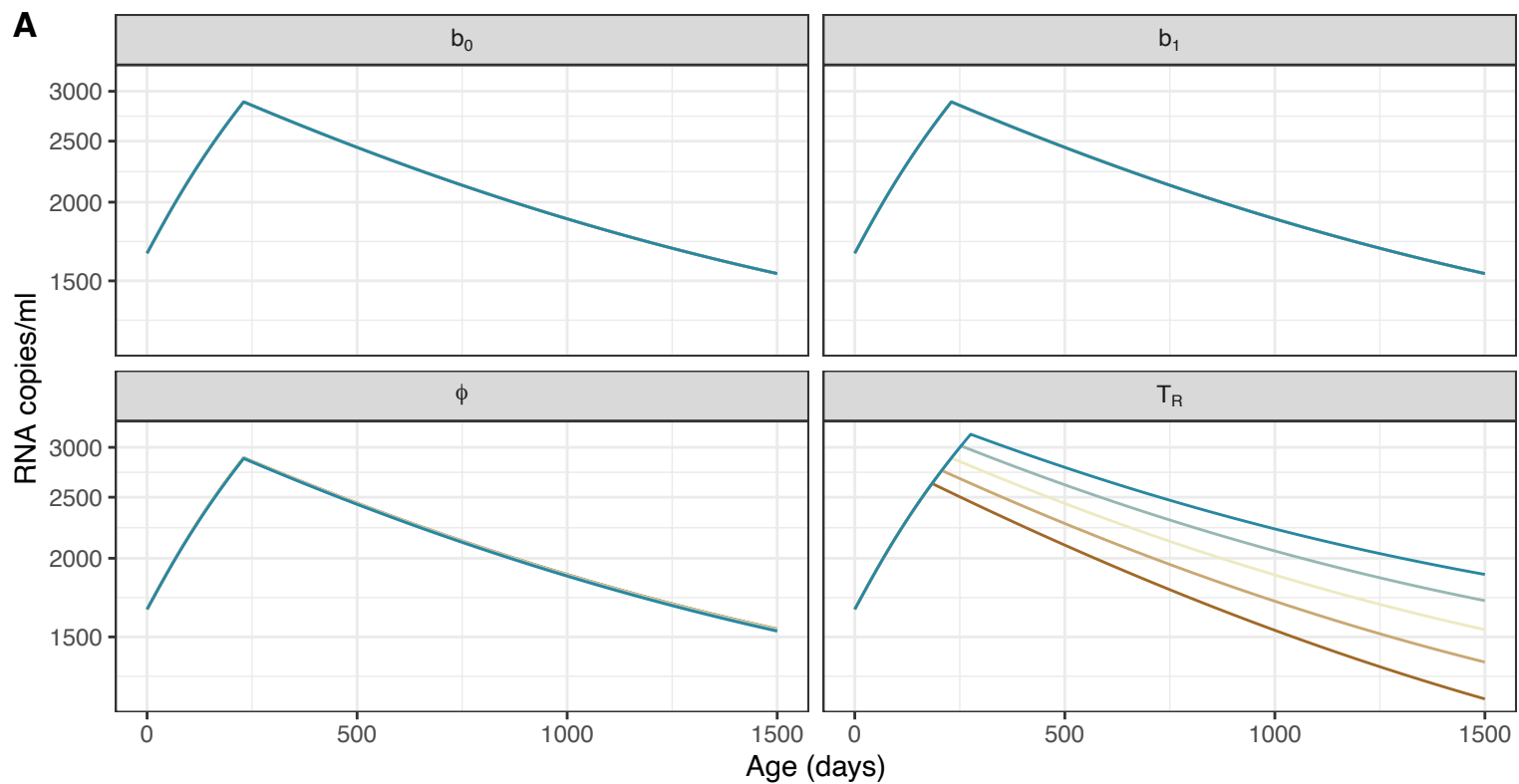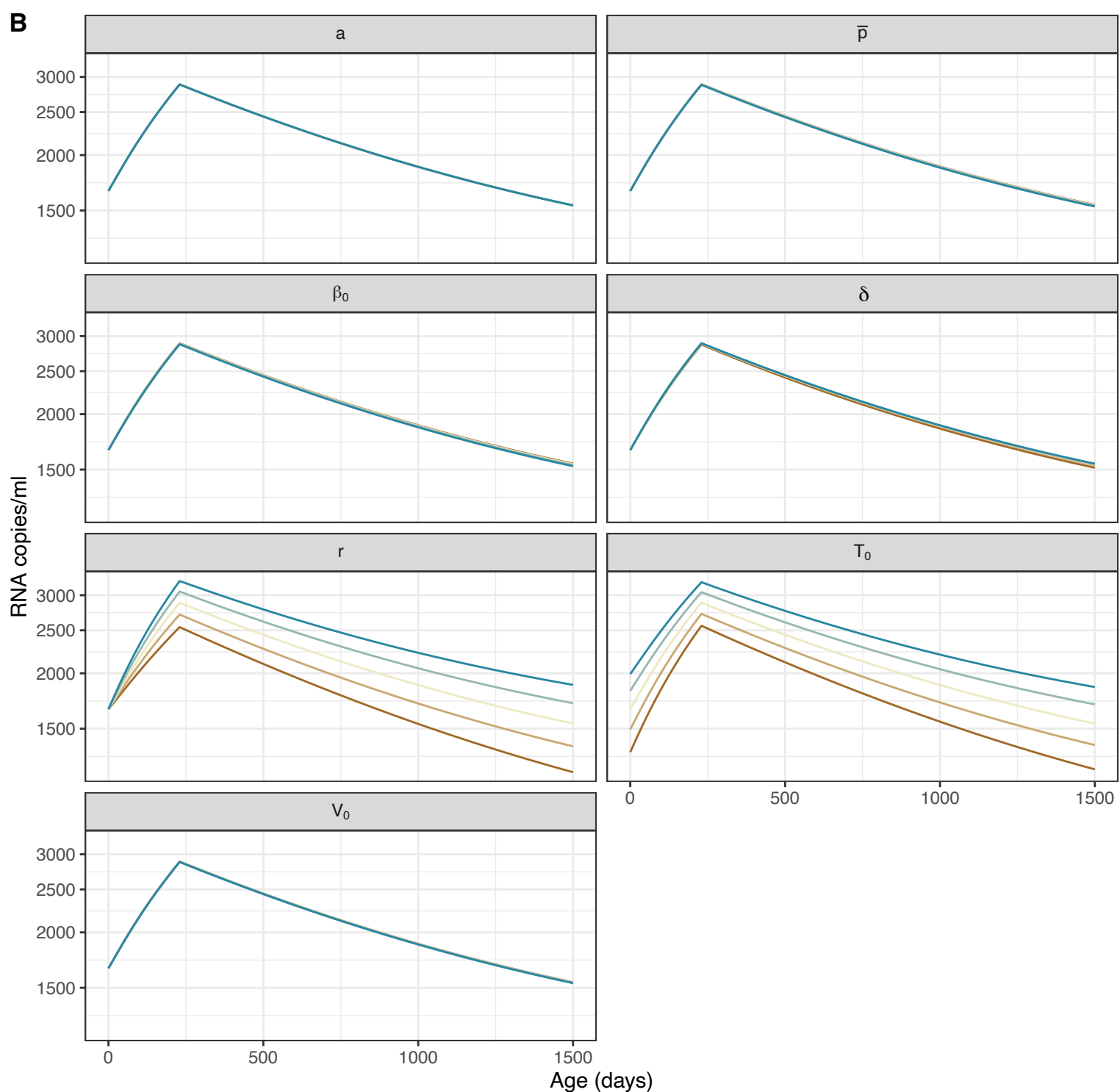

Change in estimated value — -20% — -10% — 0% — 10% — 20%

Supplement: S5 Fig — Each fixed (A) or estimated (B) parameter was varied within 20% of its original value while keeping all other parameters at their original values. Original values for the estimated parameters were the population-level means from the best-fit model. (PDF) [file ppat.1010751.s006.pdf]

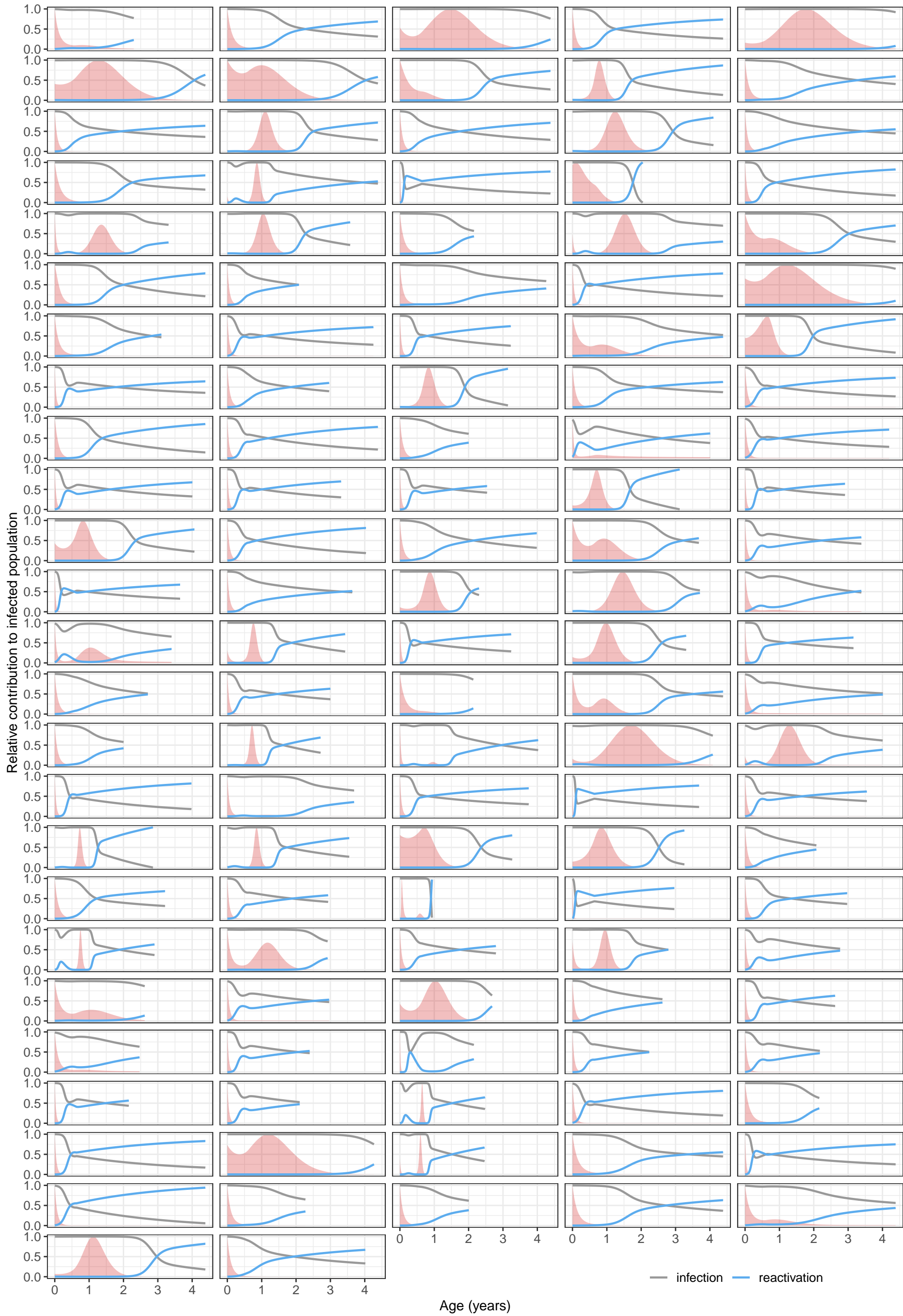

Supplement: S6 Fig — Each panel represents an infant, and red shaded regions show their VL scaled by its maximum value. (PDF) [file ppat.1010751.s007.pdf]

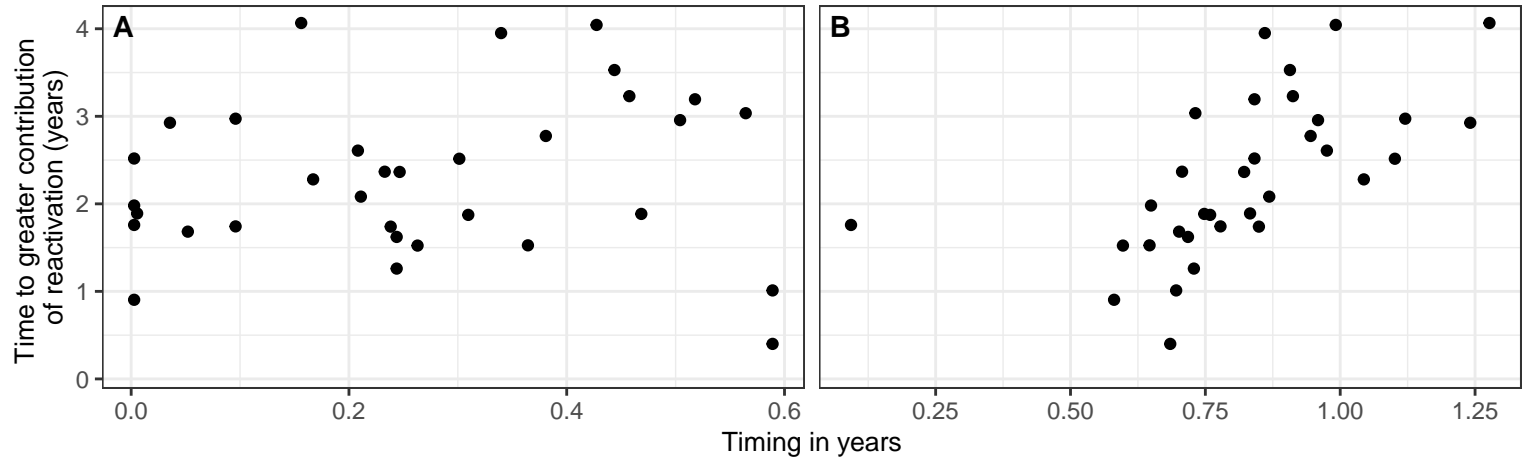

Supplement: S7 Fig — Each point represents a different infant with respect to the time at which reactivation became the major contributor to productively infected cell growth and the time at which: (A) their VL started increasing (if applicable) and (B) their VL finished increasing (if applicable); p = 0.6 and p < 0.001, respectively, and the Spearman’s rank correlation coefficient for (B) is 0.61. (PDF) [file ppat.1010751.s008.pdf]

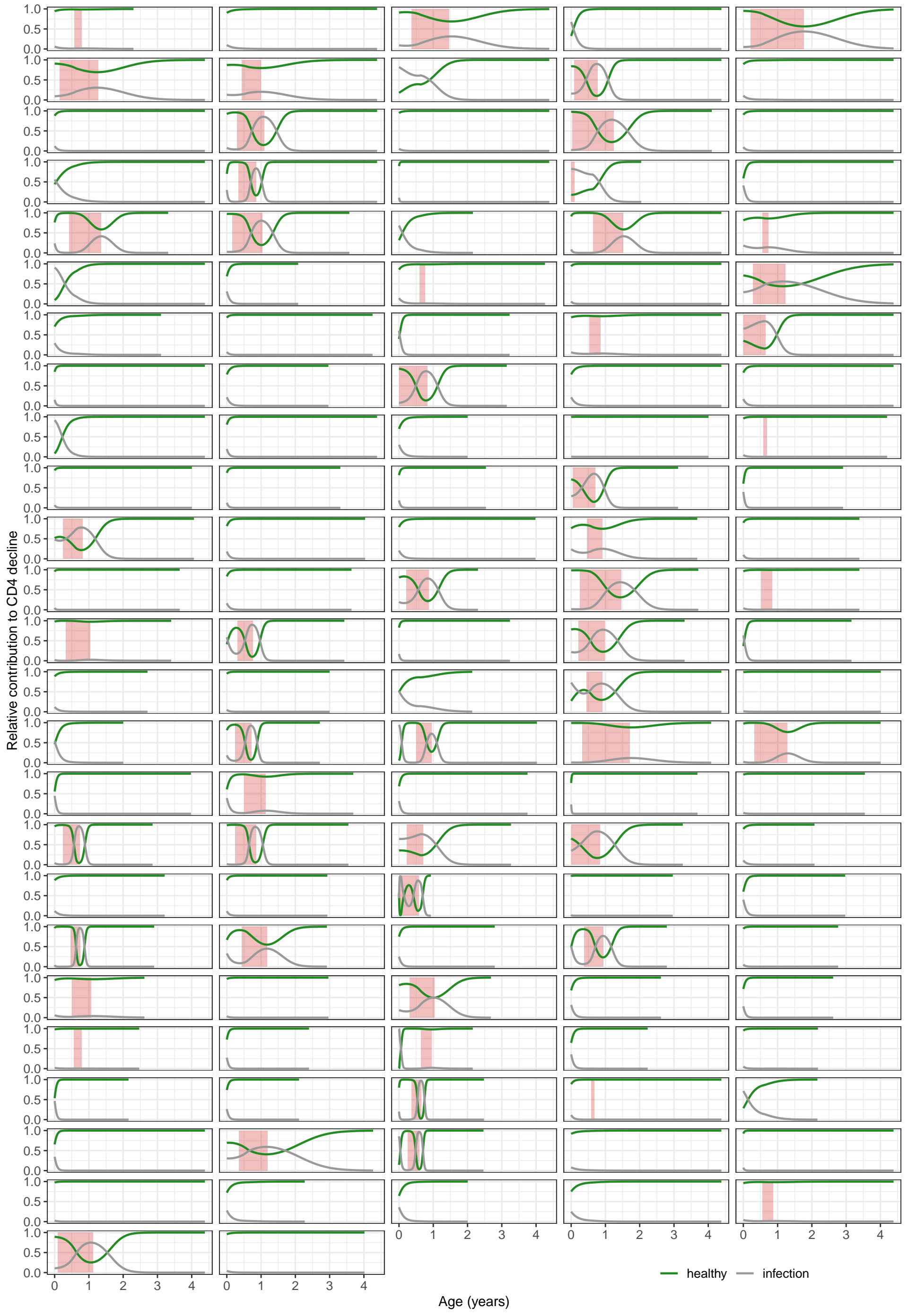

Supplement: S8 Fig — Each panel represents an infant, and red shaded regions show the periods of increasing VL (from start to peak, as shown in Fig 1B). (PDF) [file ppat.1010751.s009.pdf]

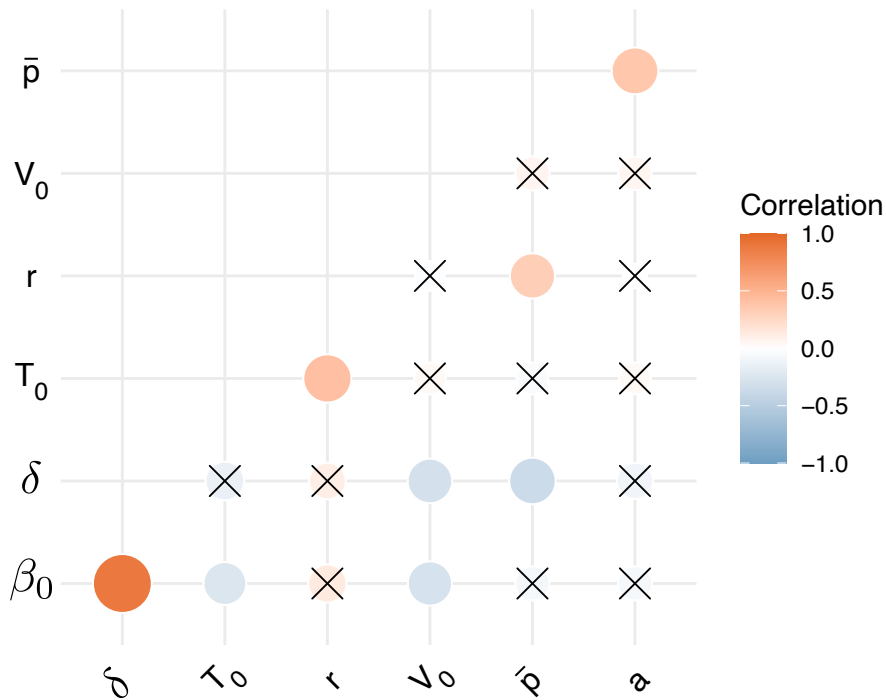

Supplement: S9 Fig — The color and magnitude of each point shows the strength of the correlation; those with p-values greater than a significance threshold of 0.05 are crossed out. p-values were adjusted using the Benjamini-Hochberg correction. The strong correlation between β0 and d was included in the nonlinear mixed effects model framework. (PDF) [file ppat.1010751.s010.pdf]

A

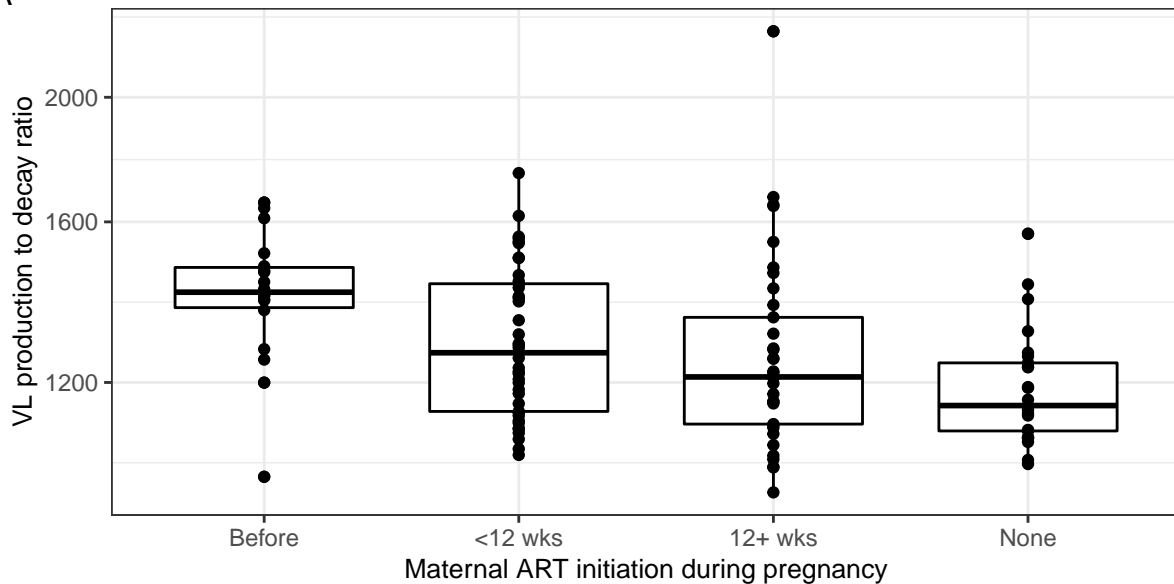

B

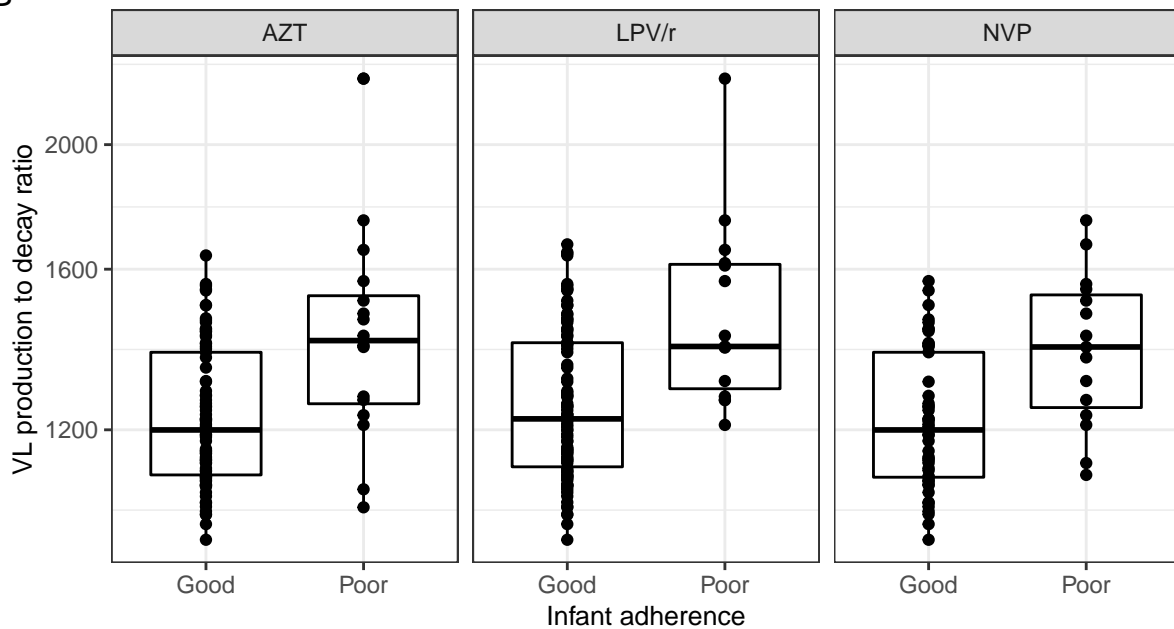

Supplement: S10 Fig — The ratio is given by p¯=p/c, in copies ml−1 cell−1. Each point represents a different infant. (PDF) [file ppat.1010751.s011.pdf]

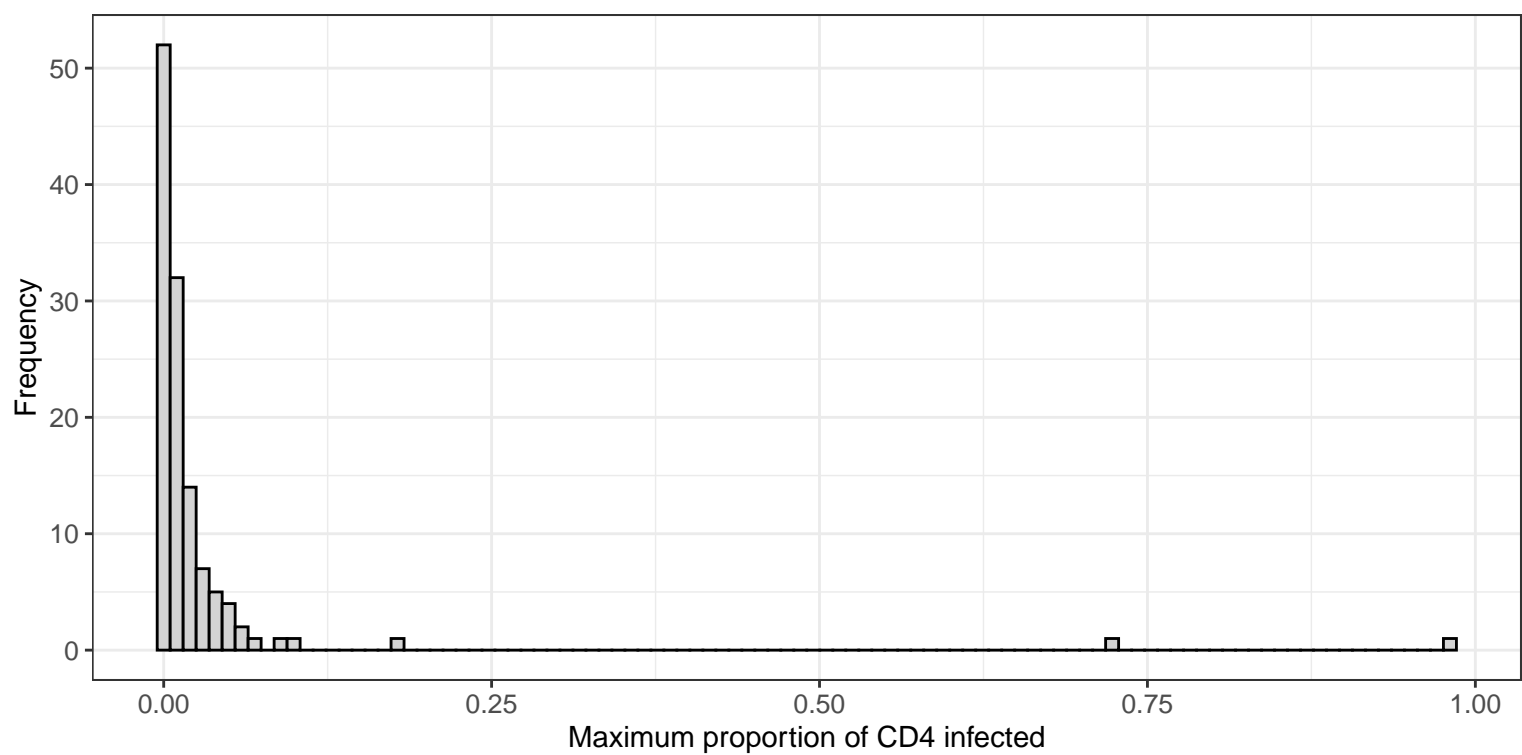

Supplement: S11 Fig — Distribution of the maximum proportion of total CD4 T cells that are infected (I(t)/(I(t)+ T(t)) across all infants. (PDF) [file ppat.1010751.s012.pdf]
