# Supplementary material for: Healthy dynamics of CD4 T cells may drive HIV resurgence in perinatally-infected infants on antiretroviral therapy
Source: PLoS Pathog. 2022 Aug 15;18(8):e1010751. doi: 10.1371/journal.ppat.1010751 (PMC9410541; doi:10.1371/journal.ppat.1010751)
Supplement: Attachment — Submitted filename: Response-to-reviewers.pdf [file ppat.1010751.s013.pdf]

We are very grateful to the three reviewers for their thoughtful comments.

In addition to the format requested by the journal we have uploaded a combined PDF of the manuscript and the SI, with all figures embedded. Major changes highlighted in blue.

Reviewer #1:

This is an interesting well-done analysis of CD4 T cell dynamics in perinatally-infected infants on ART. The authors develop a mathematical that incorporates drug adherence, the kinetics of CD4 T cell resurgence in infants on ART as well as previously described declines on CD4 T cell populations with age. They fit their new model to data from the LEOPARD study and find that their new model agrees well with the data, whereas earlier simpler models did not. The paper increases our understanding of pediatric HIV infection and the effects of early ART and introduces new equations to describe T cell dynamics in HIV-infected infants. Despite being very enthusiastic about this work, I do have some technical comments that need to be addressed.

(Minor issues)

1. In your description of the model on lines 97 and 98 you say rho is the fraction of infected cells that become productively infected or die through abortive infection. However, if they die then they should not contribute to the productively infected cells. I think you should have the abortively infected cells in the (1- rho) fraction and that you should refer to I as the productively infected cells.
2. You correctly point out that protease inhibitor blocks the production of infectious virus. Thus, shouldn't your V equation refer to infectious virus not total virus? You could then add an equation for non-infectious virus as in Perelson et al. Science 1996 and properly fit the measured RNA to the sum of infectious and non-infectious virus.

Thanks - these are good points and we revised and re-analysed the model. Beta now represents removal of target cells through either abortive or productive infection, and phi (previously rho - we changed it to distinguish it visually from p) is the proportion of these events that lead to productive infection. Latently infected cells are assumed to remain within the target population T. We feel this assumption is reasonable because they contribute to measured CD4 T cell numbers, they can presumably be re-infected to become productive, and (irrespective) we model LR reactivation as a process with total constant rate *a*, rather than by tracking the production and loss of latently infected cell numbers explicitly. As you suggest, we now also treat infectious and non-infectious virus separately, which leads to changes in the definitions of the compound parameter beta_0 and the new parameter pbar = p/c.

Our original assumption that rho (phi) is close to 1 is now incorrect, since many infection events are abortive, as Reviewer 2 pointed out. Motivated by the published estimates of phi=0.05 (Doitsh Cell 2010) and 0.35 for SHIV infections (Ke et al. J Virol 2017), we performed the fitting and model analyses again for phi = 0.05, 0.2 and 0.35.

3. To be consistent with previous literature it would be helpful to use delta for the death rate of productively infected cells rather than d.

Yes - done.

4. The compound parameter c bar is really a scaled viral production rate as suggested by your description of it as the contribution of each infected cell to the total VL. As such, why not use the symbol p bar. To avoid any confusion with the parameter representing clearance.

We agree this is better and have made the change.

5. Please explain how you decided to use a logit-normal distribution for a and normal distributions for r and TR based on your preliminary fits. Also, what was the pre-specified upper bound for a?

These choices resulted from our iterative approach to fitting in Monolix - given an initial guess at a prior, the individual parameter distributions are then evaluated for normality. We updated the priors for parameters which failed these tests. The specification of these distributions is given in the SI.

6. I was surprised that you r estimate of the death rate of infected cells was so much smaller than estimates in adults, 0.05 per day vs ~ 1 per day (cf Markowitz et al JVI 77:5037-5038 (2003). Are there other estimates of the death rate of infected cells in infants that are consistent with your estimate?

This rate is a weighted sum of rates of loss of abortively infected and short- and long-lived infected cells; we did not specifically design this study to model this process in detail. Previously we used this dataset to estimate the lifespans of short and long lived infected cell in more detail (Morris et al. JAIDS 2020) and our estimates here are consistent with the weighted average of those. We have added some references here.

7. I was also surprised that your estimate of R0 at ART initiation was 0.35 given that the median age at ART initiation was 2.5 days. If infection occurred at or near the time of birth then I would expect that on average the virus to be growing exponentially and not decaying before ART. Are there studies showing viral dynamics in untreated infants? In SHIV infection of rhesus macaques viral load increase for the first 1-2 weeks after infection – Fig 1 in JCI Insight. 2021;6(23):e152526. Please discuss this issue.

Yes this was unclear and thanks for catching it. Our estimate of R0 includes the compound parameters which subsume the ART efficacy parameters epsilon1 and epsilon2; these are not separately identifiable from the infectivity parameter beta. So it is the value of R0 after ART has taken effect. We've clarified this.

8. It is not clear how one can get access to the data used in this analysis. A data availability statement is needed

Yes. The data are now available at https://www.openicpsr.org/openicpsr/project/167981/version/V1/view

Reviewer #2

This is a very well-written paper, fitting data from young HIV-infected children with an impressive and careful mathematical fitting procedure. The mathematical model is extremely well-chosen as it is simple enough to keep parameters identifiable and rich enough to be able to account for recovery of CD4+ T cells and the natural decline in CD4 T cell counts in young children. It is impressive how well this simple model captures the wide variation in trajectories of the viral load and the CD4 count in all these children.

No major issues noted. Minor issues:

Line 104 Model: The cells undergoing abortive infection are included into the population of infected cells (that in this paper have a long expected life span). An alternative way of writing this would be to include the abortive infections in the rho parameter, i.e., cells undergoing abortive infection fail to arrive in the compartment of productively infected cells (which has been done previously). Would that make any difference, and what is the most natural way to write this?

Thank you. Reviewer #1 made the same point - please see our response there.

Line 151: Now rho=0.9999. If this were to include abortive infections it would be much smaller.

Yes indeed. We've re-done the analysis with the new value of rho (now called phi) - please see response to Reviewer #1.

Line 208: What is the R0 in the absence of ART according to these parameters? Is that a reasonable value?

See response above - we are unable to estimate R0 in the absence of ART. So we expect R0<1 here, which is what we find.

Line 218 and Fig 4B: Is there some circularity in this reasoning: children experiencing a resurgence in the VL necessarily have a slower average downslope of the VL, i.e., a lower production to decay ratio?

Yes. We have rephrased those lines to focus on the reconstitution rate as the less obvious driver of resurgence.

Lines 267-268: Is drug adherence not correlated for these 3 compounds?

Yes AZT, NVP and LPVR adherence are all correlated with one another. However, since pbar (formerly cbar) is no longer related to the adherence parameters we have removed this section.

Fig S5c: I agree this correlation is due to the outliers. Are these parametric correlations? A non-parametric test could be more careful.

(This is now Fig S6). After repeating this analysis with the refined model, the correlations (using the Spearman rank correlation coefficient) are clear. With the outlier, the correlation between time to greater contribution of the LR with time to CD4 recovery plateau is marginal ($p=0.5$); without the outlier, $p<0.05$.

Fig S9: Difficult to get a message from this, is Fig S10 not sufficient?

Yes - we have retained Figure S10 (now S1), and removed the LPV/r figure. Thanks for picking this up.

Reviewer #3

In this paper, the authors analyze a unique data set of HIV treatment in infants with a mathematical model for the viral dynamics, which also includes the dynamics of the target cells (in this case, CD4+ T cells). The paper is well written and the modeling elegantly done. The main strengths include the data set used, the novel modeling approach, and the interesting results. The paper is clearly written and the scholarship is of high caliber. Still, the work could be improved with some clarifications and also reference to some previous key publications.
Overall, the study is important in the relatively less studied field of pediatrics HIV, with focus on viral dynamics, and could be important to better understand the responses to treatment in this subgroups of infected people. The study is also somewhat novel, due to the quality of the data set and the simultaneous consideration of viral loads and CD4 counts, and treatment adherence.

Major issues (key for acceptance)

There are no extra experiments needed to complete the manuscript. However, I think that there are some important issues that would benefit from some clarification.

One of the main assumptions is implicit in the use of CD4 T-cell numbers per unit volume. Although this is common in models of viral dynamics, I wonder if the absolute changes occurring in infants are properly captured this way. For example, the CD4+ T-cell concentration (per unit volume) decreases in this age range, but the absolute number may be increasing. HIV infection may be more dependent on the total available target cells and not their concentration. At least some thoughtful discussion of this issue would be welcome.

The issue you are raising here is that changes in blood and lymphoid tissue volumes with age may impact the dynamics of cell concentrations (the rate equations will then be missing terms relating to passive changes in cell numbers or VL through changes in volume). These slow volumetric changes are taken into account in the terms in the model describing the age-dependent dynamics CD4 concentration in healthy infants (eqns 1 and 2) and operate over timescales of months. The

dynamics of cell infection, virus production and turnover are much faster and we expect changes in blood volume will not perturb these aspects of the model significantly.

Perhaps the only issue here is for the lifespans of long-lived infected cells, which may be of the order of weeks. If volume changes are occurring over these timescales, the strict interpretation of the parameter delta as an average rate of death of infected cells is lost.

Related, in part, to the previous comment, there is important work in this area that should be referenced in the current paper. See for example the papers by Lewis, Callard et al in Frontiers Immuno 2017 and in J Infect Dis 2012, among others for their treatment of CD4+ T-cell levels; and the paper by Nagaraja, Gopalan, et al Scientific Reports 2021; or the older Melvin, Rodrigo et al JAIDS 1999.

Thank you for pointing these out. We have extended our references to previous estimates of infected cell death rates, and discuss the relation the other studies of CD4 reconstitution

One of the most difficult things in a long-term study of this kind in the change in therapy protocols that occur either by medical prescription or adherence issues. It would be useful to have some discussion of what the impact of using the average adherence for each infant is, as opposed to using their time series of adherence, which might not be possible (the model would be too complex for the data). However, I must say that it was not clear at all how you actually used the adherence (even the "average adherence") of each infant in the model – do epsilon1 and/or epsilon2 (or their surrogates beta0 or c-bar) vary in time or are they estimated differently for different children? Was this used as a covariate (although it is not indicated in Table S1)? I couldn't find how you used the adherence information. The available information itself is nicely shown in figures S9 and S10, and described in the first section of the supplementary material. But how was it used in the modeling?

Each child was allowed a different and constant value of the compound parameter Beta0, which (in our revised model) includes the transmission rate beta as well as the two efficacy parameters epsilon1 and epsilon2. As you point out, adherence likely fluctuates; and yet the approximation of an average, constant level gives surprising explanatory power. As a (weak) form of out-of-sample prediction, we then looked for associations between model parameters and the binary, time-averaged adherence readout derived from questionnaires. We now emphasise in Results that this adherence measure was not included as a covariate in the model.

It is not clear what is the impact and the need to consider the latent reservoir, namely the inclusion in the model of rho and a. Can you fit the data when rho= 1 and/or when a= 0?
Related with the previous comment, you state in line 199 that "in almost all infants the importance of new infection events was eventually superseded by reactivation from the latent reservoir". However, this is only true when there is no more measurable virus (in the next sentence it even says that "this displacement was delayed by viral resurgence events…"). So, how relevant is the infection from the latent reservoir in the context of your study. Are these needed only for virus rebound when treatment is stopped?

The model fits with a=0 were very poor, with a very large AIC difference (Table 2). We therefore rejected these models; reactivation of latently infected cells is required to explain these data, especially at late times after ART initiation.

One of your findings is that longer duration of maternal prenatal ART was associated with larger production of virus and with viral resurgence. This seems counter intuitive, and a little more discussion would be helpful. Is it that longer treatment may lead to transmission of resistant strains? In the discussion (line 286), you do say that longer prenatal ART is associated with larger viral reservoir in the infants. This is perhaps more puzzling that explanatory.

Yes, this is indeed surprising and needed more said. We've extended the paragraph in the Discussion on maternal ART effects, to include some speculation on the basis of this result.

In your final conclusion, you state that resurgence may be inevitable in certain parameter regimes. This was not clear. What about increasing the adherence or improving efficacy of the drug protocol? The latter is a composed parameter, so perhaps you can't check in your model. But you could simulate the first effect for a couple of infants with resurgence to check what would happen assuming perfect adherence and keeping other parameters the same.

If one sets beta0 to zero (100% adherence) then resurgence does not happen - all one sees is non-infectious virus generated at a low and constant rate by LR reactivation. As you point out, because adherence/efficacy are embedded within the single parameter beta0, we're not able to meaningfully simulate the effect of partially increasing adherence.

Minor issues:

Line 60: interquartile range is formally a single number, the range, although I do recognize that it is often used in the way presented here, i.e., the 25th and 75th percentiles.

Corrected.

Line 111: Using c-bar is not a great choice, because it gives the impression that this parameter is related to c (the clearance rate), but in fact it is inversely proportional to c, and you interpret it as the "contribution of each infected cell to the total viral load", so more like p (production rate). It seems that p-bar would be easier for the reader.

Good point, also raised by other reviewers. We've changed this.

Line 145: please add the word "structural" to your description of "We confirmed the identifiability of all parameters…".

Done.

Table 1 (and also in some parts of the text, e.g., line 206): you present the "SE= standard error (random effect)". But Monolix usually (by default) presents the SE of the fixed effect, and the standard deviation of the random effect. Please confirm which one of these you are discussing, or if

indeed it is the standard error of the random effects, which is something different and indeed probably not very appropriate for this situation.

Thank you for pointing this out. We are indeed presenting the standard error of the fixed effect in Table 1 and have corrected the caption.

Line 153: You used the general AIC formula. Usually, it is recommended to use the small sample corrected AIC, even when the sample seems large enough, because you are also estimating many parameters (in Table 1, 19 parameters or so, including error and correlation parameters). However, technically the small sample AIC formula does not apply directly to multivariate models (and fits), as is the case here and further corrections are needed. See for example the book by Burnham and Anderson (section 7.7.6). This is just something for you to consider what is the best option in this case.

Thank you for this. Given this uncertainty, and that the AIC differences are so large (all >40 and most in the hundreds), and that the alternative model fits are so obviously inferior, we retain the use of AIC for simplicity.

Figures with time courses: I suggest that you plot the figure with the line on top of the data, rather than the data on top of the line. The latter makes it difficult to see the fits when there is a concentration of data points.

Done.

Line 219: what do you mean with "deterministic model parameters", deterministic as opposed to what?

We were attempting to contrast deterministic resurgences predicted by this ODE model to ones resulting from stochastic reactivation events. We've removed "deterministic" here.

Figure 4: Please define the labels in the x axis: "Before" - before being pregnant? Define "12+ wks" more than twelve weeks of treatment or initiated at more than 12 weeks of pregnancy? This only become clear when consulting table S1.

Thanks for picking this up - we've clarified.

Figure 4 caption: description of panel (F), the words "is associated" seem extra.

Done.

Line 310: You state that "frequency of infection in CD4 T cells is usually small", but was this true in your case. That is, what is the time course of I(t)/(T(t)+I(t)) in your results?

This was indeed true. Only 2/122 infants had a maximum frequency of infection greater than 0.2 and only 10/122 greater than 0.05. We've added an SI figure showing the distribution of maximum frequencies of infection.

Table S1: It is not clear that "Variable treatment: continuous" means when you describe the variable as grouped. For example, "Birth weight" was divided into two groups more or less than 2500g. How do you treat this as a continuous covariate in Monolix? The same question for "Mother's viral load", etc…

Variable treatment refers to how the variable was considered in statistical analysis when testing for associations between resurgence, model parameters and clinical covariates. We did not include any of these clinical covariates in the Monolix fitting procedure.

For continuous covariates, the groups presented (e.g. Birthweight < or > than 2500g) are provided to give the reader an idea of their distribution in this cohort but are not used in the analysis. We now clarify (footnote in the table within S1 Text).

Figure S7: The symbols seem to have different sizes, but this is not described in the caption.

The size is simply the (absolute) strength of the correlation. We have clarified this in the figure caption.
