# Supplementary material for: Healthy dynamics of CD4 T cells may drive HIV resurgence in perinatally-infected infants on antiretroviral therapy
Source: PLoS Pathog. 2022 Aug 15;18(8):e1010751. doi: 10.1371/journal.ppat.1010751 (PMC9410541; doi:10.1371/journal.ppat.1010751)
Supplement: Attachment — Submitted filename: Morris et al review2.docx [file ppat.1010751.s014.docx]

The authors addressed all the concerns I raised in my previous review. I have only one very minor comment about the revision. On p4, where epsilon_1 and epsilon_2 are defined the authors need to make sure that for both epsilons, epsilon<1 reflects both incomplete drug adherence and the inability of drug therapy to completely block reverse transcriptase inhibitor or protease inhibitor activity. Currently, the authors define epsilon_1 <1 as implying only incomplete adherence and epsilon_2<1 as only implying less than 100% effective protease inhibitor activity.
