# Supplementary material for: Healthy dynamics of CD4 T cells may drive HIV resurgence in perinatally-infected infants on antiretroviral therapy
Source: PLoS Pathog. 2022 Aug 15;18(8):e1010751. doi: 10.1371/journal.ppat.1010751 (PMC9410541; doi:10.1371/journal.ppat.1010751)
Supplement: Attachment — Submitted filename: Corrections-V2.rtf [file ppat.1010751.s015.rtf]

We have addressed all of the remaining reviewer comments:


The authors addressed all the concerns I raised in my previous review. I have only one very minor comment about the revision. On p4, where epsilon_1 and epsilon_2 are defined the authors need to make sure that for both epsilons, epsilon<1 reflects both incomplete drug adherence and the inability of drug therapy to completely block reverse transcriptase inhibitor or protease inhibitor activity.  Currently, the authors define epsilon_1 <1 as implying only incomplete adherence and epsilon_2<1 as only implying less than 100% effective protease inhibitor activity.

Thanks for picking this up. We have clarified as suggested (lines 90-98)

1) Page 9, line 231: I believe this should be "standard error of the fixed effects" and not random effects. Please re-check throughout.

yes - corrected

2) Page 9, line 234: are these the limits of the interval, or the range of multiple intervals across the children.

Percentiles of the distribution of point estimates across the cohort of children. We now clarify.

3) Page 9, line 258: instead of p, it would me more appropriate to say "not significant at the alpha=0.05 level."

Corrected

4) Page 15, line 348: regarding your estimate of 16 days, there is a new paper that may be relevant, see White et al. PNAS 2022: https://doi.org/10.1073/pnas.2120326119

Thank you - we now reference this (line 351)
